# Study on Aging Mechanism and High-Temperature Rheological Properties of Low-Grade Hard Asphalt

**DOI:** 10.3390/ma16165641

**Published:** 2023-08-16

**Authors:** Liang Song, Xiaodong Xie, Pengcheng Tu, Jingjing Fan, Jie Gao

**Affiliations:** 1 Xinjiang Transportation Investment (Group) Co., Ltd., Urumqi 830006, China; slpl@sohu.com; 2 Xinjiang Transportation Investment Construction Management Co., Ltd., Urumqi 830099, China; 3 Key Laboratory of China’s Transportation Industry for Highway Engineering Technology in Arid Desert Region, Urumqi 830099, China; 4 Xinjiang Vocational and Technical College of Communications, Urumqi 831401, China; xiaodong3056@163.com; 5 School of Transportation and Logistics, Xinjiang Agricultural University, Urumqi 830091, China; 320212738@xjau.edu.cn (P.T.); 320222569@xjau.edu.cn (J.F.); 6 School of Civil Engineering and Architecture, East China Jiaotong University, Nanchang 330013, China

**Keywords:** low-grade hard asphalt, high-temperature rheological properties, thermal oxygen aging, UV aging

## Abstract

In order to investigate the potential application of low-grade hard asphalt in high-temperature and high-altitude areas, various tests were conducted to analyze the performance and high-temperature rheological properties of 30#, 50#, and 70# matrix asphalt under thermo-oxidative aging and ultraviolet aging. The tests utilized for analysis included the examination of basic asphalt properties, Fourier transform infrared spectroscopy (FTIR), atomic force microscopy (AFM), gel permeation chromatography (GPC), dynamic shear rheology (DSR), and multi-stress creep recovery (MSCR). The results indicate a progressive decrease in asphalt performance with increasing aging time. Prolonged exposure to thermal oxygen aging and ultraviolet irradiation significantly diminishes the plasticity of asphalt. The carbonyl index and sulfoxide index of asphalt increase after thermal oxygen aging and ultraviolet aging. Notably, 30# asphalt demonstrates greater resistance to aging compared to 50# and 70# asphalt under long-term high ultraviolet radiation. The LMS% of 30#, 50#, and 70# asphalt increases by 14%, 15%, and 16%, respectively. Following photothermal oxidative aging, a larger proportion of lighter components in the asphalt transforms into resins and asphaltenes. The high-temperature rheological properties of the three types of asphalt rank as 30# > 50# > 70#, while within the same type of asphalt, the high-temperature rheological properties rank as PAV > UV3 > UV2 > UV1 > RTFOT > virgin. Elevating temperature, stress level, and stress duration negatively impact the high-temperature stability of asphalt. In general, low-grade asphalt demonstrates superior anti-aging ability and high-temperature rheological properties during the aging process.

## 1. Introduction

High-temperature and high-altitude regions are characterized by aridity, intense heat, and high levels of ultraviolet radiation. These conditions contribute to severe rutting issues in asphalt pavements within such areas [1,2]. To address the anti-rutting requirements of pavements, high-content styrene-butadiene-styrene (SBS)-modified asphalt is commonly employed, or anti-rutting agents are added for asphalt modification. While this approach partially mitigates high-temperature rutting, the preparation process for modified asphalt is intricate and costly. In addition to polymer-modified asphalt, low-grade hard asphalt exhibits favorable resistance to high-temperature rutting and offers a simpler production process without the need for additives or modifiers [3,4]. Tong et al. [5,6] conducted a comparative study on 70# asphalt, revealing that 30# hard-grade asphalt demonstrates excellent resistance to high-temperature deformation and exhibits good water stability. This underscores the effectiveness of utilizing low-grade hard asphalt in reducing asphalt pavement rutting and enhancing pavement durability.

Asphalt, as an organic polymer material, is susceptible to aging when exposed to heat, light, and oxygen [7,8]. The performance of asphalt can be altered by factors such as increased traffic load and environmental influences, consequently impacting the durability of asphalt pavement under high temperatures. When assessing the macroscopic properties of asphalt before and after aging, penetration, softening point, ductility, viscosity, and rheological properties are commonly employed to characterize the effects of aging [9,10]. Studies conducted by Li [11] and Cao et al. [12] demonstrate the impact of RTFOT, PAV, and UV aging tests on the fundamental properties of asphalt. Several researchers argue that analyzing the high-temperature performance of asphalt through its rheological properties is more scientifically rigorous [13,14]. The dynamic shear rheological (DSR) test is utilized to investigate the high-temperature rheological properties of asphalt, while the rutting factor (G*/sinδ) serves as an indicator for evaluating its high-temperature performance [15,16]. Moreover, some scholars propose the multi-stress creep recovery (MSCR) test to effectively assess the rheological properties of asphalt, with its high-temperature performance evaluated through parameters such as creep recovery rate and non-recoverable creep compliance [17,18].

Explaining the aging mechanism of asphalt solely through macroscopic tests presents challenges, as the four components, functional groups, molecular weight, and microstructure of asphalt significantly influence its performance [19]. Consequently, there is considerable research interest in analyzing the aging mechanism of asphalt through microscopic performance tests [20]. Following asphalt aging, the proportions of its four components undergo significant changes [21]. Volatilization occurs in saturates and aromatics, leading to an increase in the proportion of resins and asphaltenes, with some resins converting into asphaltenes during aging [22]. Fourier transform infrared spectroscopy (FTIR) is the most commonly employed method for characterizing asphalt aging [23]. Researchers explored the aging mechanism by analyzing changes in molecular structure and chemical bonds observed in the infrared spectrum [24]. They also proposed various index changes, such as the carbonyl index, sulfoxide index, aromatic index, and butadiene index, as means to evaluate the degree of asphalt aging. Among these indices, the carbonyl and sulfoxide indexes are particularly regarded as relevant indicators of asphalt aging [25,26]. In another study, the effect of aging on asphalt roughness was investigated using atomic force microscopy (AFM) [27,28]. The findings revealed that the microscopic surface roughness of asphalt gradually increased with the progression of aging.

Low-grade hard asphalt offers several advantages, such as high rigidity, hardness, and resistance to rutting, making it a suitable choice for pavement materials in high-temperature and high-altitude areas. However, aging contributes to the deterioration of asphalt performance significantly. Currently, there is a limited body of research on the aging mechanism and rheological properties of low-grade hard asphalt. Therefore, this study focuses on 30# and 50# low-grade matrix asphalts, comparing them with 70# matrix asphalt. Through tests examining basic properties, microscopic characteristics, and dynamic shear rheology, the study demonstrates the aging attenuation patterns and performance changes of low-grade asphalt in high-temperature and high-altitude environments. The findings provide practical suggestions and technical support for the application of low-grade asphalt in high-temperature and high-altitude areas.

## 2. Materials and Methods

### 2.1. Raw Material

Karamay 30#, Kunlun 50#, and Kunlun 70#, manufactured by China National Petroleum Corporation in Karamay, were chosen as the virgin asphalt specimens for the tests. These specific asphalts were locally produced in Xinjiang. Table 1 presents the basic parameters of the three types of asphalt.

### 2.2. Aging Method

**Figure 1:**
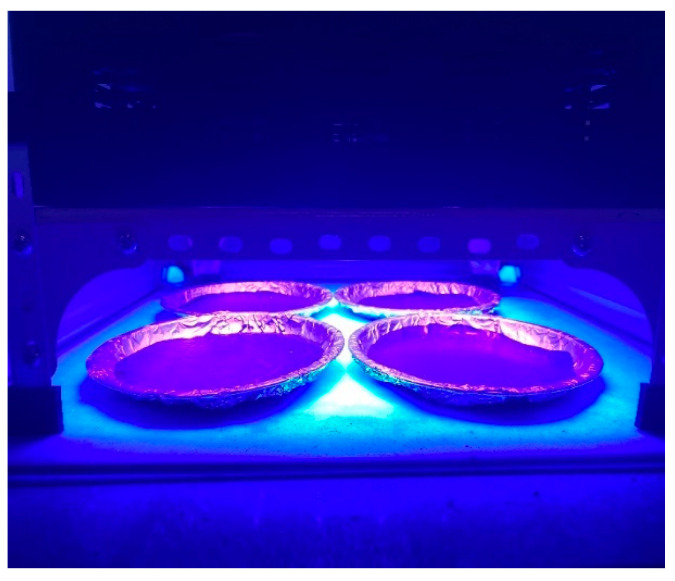
The environment simulation equipment of UV aging.

Three aging methods were employed to simulate the aging conditions of low-grade asphalt in high-temperature and high-altitude areas. These methods include short-term aging, long-term aging, and ultraviolet aging. In the short-term aging simulation test, the rotating thin film oven test (RTFOT) method was utilized to replicate the aging resulting from asphalt oxidation reactions during storage, transportation, mixing, and paving. For the long-term aging simulation, a pressure aging vessel (PAV) tester was used to imitate the oxidative aging of asphalt during road usage. Ultraviolet aging was conducted by exposing asphalt samples to ultraviolet LED lamp beads. The lamp beads were positioned 10 cm away from the samples, with an ultraviolet radiation intensity of 300 mW/cm^2^, a sample surface temperature of 70 °C, and an ultraviolet wavelength of 395 nm, as illustrated in Figure 1. Previous research findings [29] indicate that ultraviolet radiation can penetrate the asphalt film to a thickness of approximately 1 to 3 mm. In this experiment, the asphalt film thickness was controlled at 1 mm by adjusting the quality of the asphalt sample. After undergoing the RTFOT test, the asphalt samples were subjected to indoor ultraviolet irradiation for 32 h, 64 h, and 96 h, corresponding to aging levels equivalent to 1, 2, and 3 years in the local environment, respectively. These samples are labeled as UV1, UV2, and UV3, respectively.

### 2.3. Experimental Methods

#### 2.3.1. Basic Physical Indices

The physical properties of asphalt primarily encompass penetration at 25 °C, softening point, and ductility at 15 °C. These properties were determined following the procedures outlined in the ASTM D-5, ASTM D-36, and ASTM D-113 standards.

#### 2.3.2. FTIR Test

A Thermo Scientific Nicolet iS20 spectrometer was utilized to record the spectra of asphalt before and after aging. The test range covered 600−4000 cm^−1^, with a resolution of 4 cm^−1^ and a total of 32 scans. The aging analysis of the three types of asphalt involved the assessment of the carbonyl index and sulfoxide index, which were calculated using Equations (1) and (2), respectively [30]. Three samples were randomly selected from each asphalt for FTIR testing, and the average of the three samples was taken as carbonyl index and sulfoxide index.

(1) $$I_{c=o}=\frac{A_{c=o}}{\sum A_{2000\;-\;600}}$$

(2) $$I_{s=o}=\frac{A_{s=o}}{\sum A_{2000\;-\;600}}$$

In the formula, *A*_C=O_ is the area of the carbonyl peak; *A*_S=O_ is the area of the sulfoxide peak; and $\sum A_{2000\;-\;600}$ is the sum of the area of each peak in the range of 2000 to 600 cm^−1^.

#### 2.3.3. GPC Test

In this experiment, a PL-GPC50 gel permeation chromatograph was employed. The column length was 300 mm, consisting of a total of 3 columns. The solvent used was tetrahydrofuran, with a flow rate of 1.0 mL/min. The concentration of the sample solution was set at 2.0 mg/mL.

#### 2.3.4. AFM Test

A Bruker Dimension ICON AFM instrument was utilized to scan various asphalt samples in tapping mode. An RTESPA-150 probe was employed for the test, with an elastic coefficient of 5 N/m and a resonance frequency of 150 kHz. The scanning area covered 20 × 20 μm.

#### 2.3.5. DSR Test

The temperature of the asphalt was measured using a Kinexus dynamic shear rheometer. For the temperature scanning test, a parallel plate with a diameter of 25 mm and a plate spacing of 1 mm was employed. The fixed loading frequency was set at 10 rad/s. The test range spanned from 52 °C to 82 °C, with a test interval of 6 °C [31].

#### 2.3.6. MSCR Test

The loading process of the MSCR test consisted of two stages. In the first stage, the asphalt sample underwent a 1 s creep deformation and a 9 s recovery deformation under a stress condition of 0.1 kPa. This process was repeated for a total of 10 cycles. In the second stage, the asphalt sample experienced creep deformation for 1 s under a stress condition of 3.2 kPa, followed by a 9 s recovery deformation. This stage was also repeated for a total of 10 cycles. The test temperatures employed were 52 °C, 64 °C, 72 °C, and 82 °C.

## 3. The Aging Mechanism of Low-Grade Hard Asphalt

### 3.1. Aging Characteristics of Low-Grade Asphalt Based on FTIR

#### 3.1.1. Infrared Spectral Characteristics of Asphalt before and after Aging

**Figure 2:**
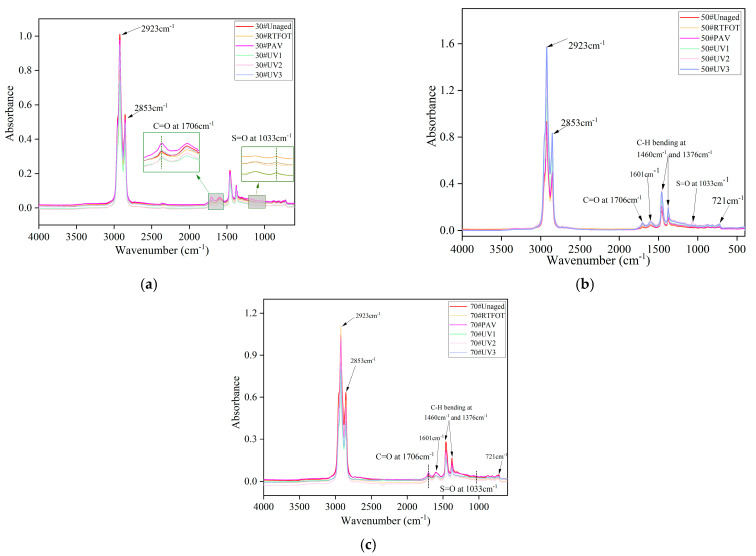
Fourier transform infrared spectroscopy of asphalt: (**a**) 30# asphalt; (**b**) 50# asphalt; (**c**) 70# asphalt.

Figure 2 illustrates the FTIR spectra of 30#, 50#, and 70# asphalt before and after short-term aging, long-term aging, and ultraviolet aging. In the functional group region, strong absorption peaks can be observed at 2923 cm^−1^ and 2853 cm^−1^, corresponding to C-H bond stretching vibrations. In the fingerprint region, relatively prominent absorption peaks are present at 1460 cm^−1^ and 1376 cm^−1^. The peak at 1460 cm^−1^ corresponds to the in-plane stretching vibration of C-H bonds in CH2 groups, while the peak at 1376 cm^−1^ corresponds to the stretching vibration of CH3 groups. The positions of the absorption peaks in the infrared spectra of the three asphalt grades remain roughly the same under different aging methods, with similar spectral bands appearing. However, the relative intensity of the absorption peaks varies among the three grades of asphalt. Overall, the absorbance is highest for 50# asphalt, followed by 70# and then 30#. From Figure 2a, it is evident that regardless of thermo-oxidative aging or ultraviolet aging, the peak areas at 1706 cm^−1^ and 1033 cm^−1^ significantly increase for 30# asphalt, while the peak areas at 2923 cm^−1^ and 2853 cm^−1^ decrease. This indicates an increase in carbonyl and sulfoxide groups and a decrease in alkyl groups. During the aging process of asphalt, carbon–carbon double bonds break and combine with oxygen, leading to the formation of polar functional groups, such as carbonyl and sulfoxide groups. These polar functional groups further reorganize and alter the composition of the asphalt, resulting in changes in its performance [29].

#### 3.1.2. Carbonyl and Sulfoxide Index of Asphalt before and after Aging

**Figure 3:**
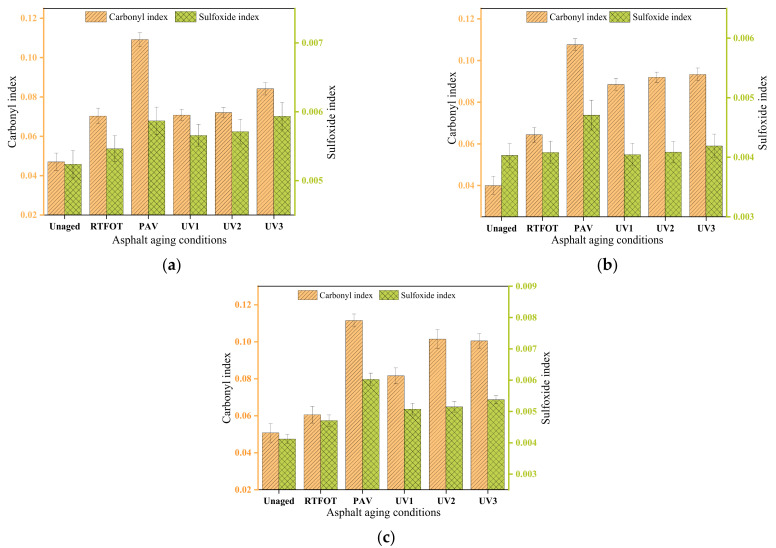
The change of functional group index: (**a**) 30# asphalt; (**b**) 50# asphalt; (**c**) 70# asphalt.

To further investigate the impact of aging on the carbonyl and sulfoxide groups of asphalt, the carbonyl index and sulfoxide index were utilized for analysis. The calculation results are presented in Figure 3.

Figure 3 clearly indicates that both the carbonyl index and sulfoxide index of asphalt exhibit increases after thermal oxygen aging and ultraviolet aging. The carbonyl index demonstrates a significant growth rate, with the functional group index increasing along with the asphalt grade. In terms of thermal oxygen aging, the carbonyl index of 30#, 50#, and 70# asphalts after short-term aging exhibits increases of 49.8%, 61%, and 19.3%, respectively compared to unaged asphalt. After long-term aging, the carbonyl index increases by 133%, 169%, and 120% for 30#, 50#, and 70# asphalts, respectively. Notably, the short-term aging effect is less pronounced for 70# asphalt, whereas the long-term aging effect is substantial. Regarding the sulfoxide index, the growth rates for 30# and 50# asphalt are less than 15%, while the short-term and long-term aged 70# asphalt experiences increases of 14.4% and 46.5%, respectively. During the ultraviolet aging stage (UV1 to UV3), there is minimal change in the carbonyl index and sulfoxide index for the three grades of asphalt. Specifically, the carbonyl index of 30#, 50#, and 70# asphalt increases by 18.9%, 5.5%, and 23.2%, respectively, from UV1 to UV3 aging. Similarly, the sulfoxide index increases by 5.0%, 3.7%, and 5.9%, respectively. These findings highlight that the UV aging degree is most apparent for 70# asphalt.

The variation range of the carbonyl index and sulfoxide index during the thermo-oxidative aging stage is greater than those observed during the ultraviolet aging stage. This suggests that ultraviolet aging, to some extent, inhibits the occurrence of oxidation reactions. Previous studies [29] have indicated that ultraviolet light can only induce aging in the top 4.5 μm of asphalt, while the lower layers of asphalt are aged through the diffusion of the surface-aged asphalt. This process also results in a decrease in the oxidation reaction rate of the asphalt. As a result, in the long-term thermal oxygen aging and ultraviolet aging processes, the functional group index of 30# asphalt is lower than those of 50# and 70# asphalt. It can be inferred that under conditions of high ultraviolet radiation, 30# asphalt exhibits greater resistance to aging compared to 50# and 70# asphalt.

**Figure 4:**
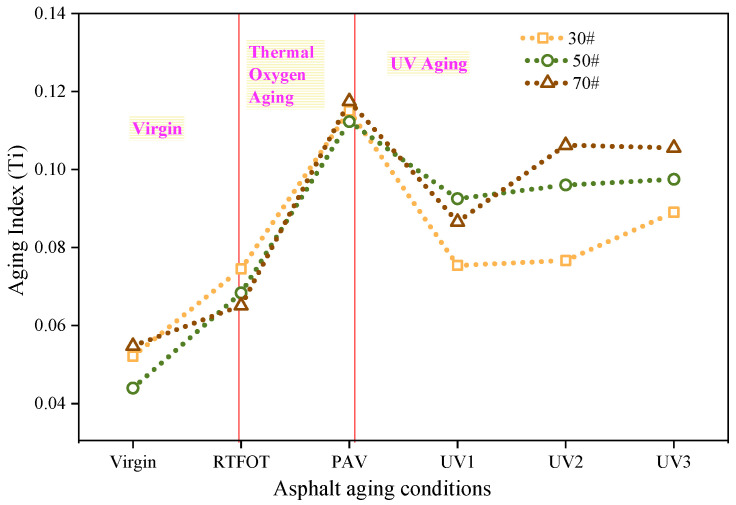
Asphalt aging index Ti.

To further examine the combined effect of the carbonyl and sulfoxide indexes on the aging performance of asphalt, this study utilizes the total index (Ti) proposed by Qian et al. [32]. The results are presented in Figure 4. During the thermo-oxidative aging stage, the aging trends of 30# and 50# asphalt are similar. The Ti indexes for both asphalts increase by 45.2% and 55.5%, respectively, from non-aging to short-term aging. Furthermore, from short-term aging to long-term aging, the Ti indexes increase by 51.9% and 64.2%, respectively, for the two asphalts. For 70# asphalt, the Ti index increases by 18.9% after short-term aging and by 80.4% after long-term aging. These findings indicate that the short-term aging performance of 70# asphalt is better, while the long-term aging performance is poorer.

During the ultraviolet aging stage, the Ti indexes of all three types of asphalt gradually increase with aging time. Furthermore, from UV1 to UV3 aging, the Ti indexes for 30#, 50#, and 70# asphalts increase by 18.1%, 5.4%, and 21.9%, respectively. These results indicate that the thermo-oxidative aging performance undergoes the most significant change for 70# asphalt, while 50# asphalt exhibits the most stability. In general, 30# and 50# asphalts demonstrate better durability than 70# asphalt in high ultraviolet radiation environments.

### 3.2. Aging Characteristics of Low-Grade Asphalt Based on AFM

**Figure 5:**
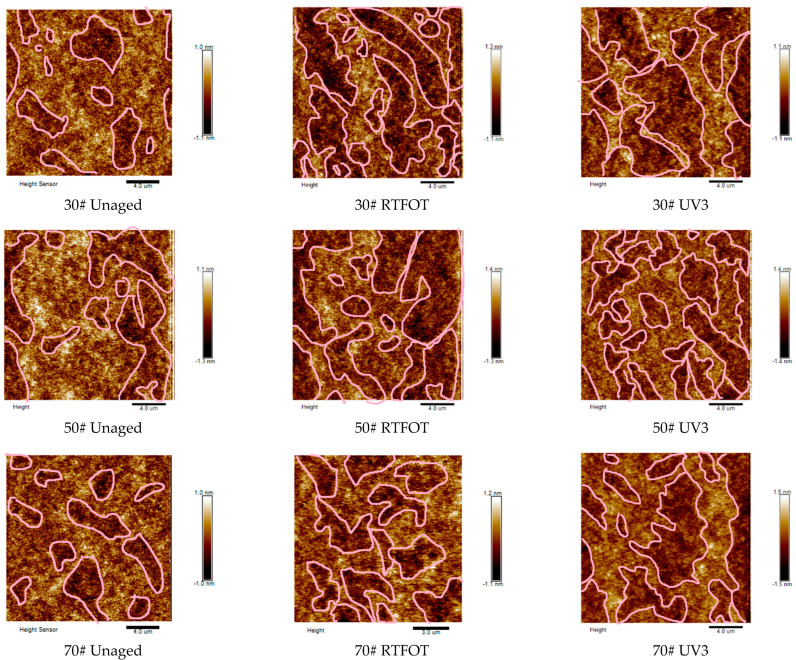
AFM Elevation map of asphalt.

To investigate the aging characteristics of asphalt during the photothermal oxidative aging process, AFM elevation images of the three types of asphalt were analyzed before and after short-term thermo-oxidative aging and long-term ultraviolet aging using AFM, as depicted in Figure 5. The microscopic surface of the unaged asphalts exhibits light and dark substances. Following short-term aging, the presence of “black substances” in the images becomes more pronounced. This can be attributed to an increase in heavy components within the asphalt due to thermo-oxidative aging. During the photothermal oxidative aging process, the action of photothermal oxygen reduces the content of saturates and converts certain small molecular components into molecules with higher polarity. For instance, aromatics are transformed into resins, which subsequently convert into asphaltenes. Additionally, aromatics and resins associate with one another and accumulate together, leading to an increase in the molecular weight of the asphalt and a rise in heavy components.

**Figure 6:**
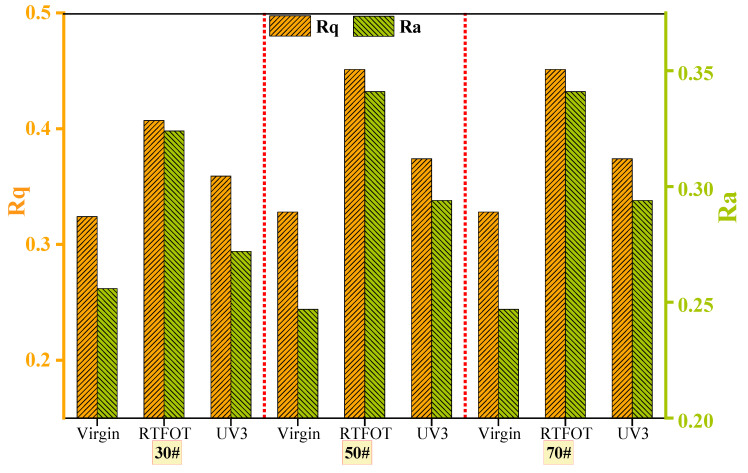
Surface roughness of asphalt before and after aging.

To quantitatively characterize the change in surface roughness of each asphalt material, the root mean square of height (Rq) and the average height (Ra) were employed as evaluation indices. The results are presented in Figure 6. It is observed that the roughness values of the three unaged asphalts are very similar. After short-term aging, the roughness index increases. Specifically, the root mean square values of 30#, 50#, and 70# asphalts increase by 25.62%, 27.27%, and 62.08%, respectively, compared to the original asphalt. The roughness of 70# asphalt exhibits the most significant change. However, following UV aging, the roughness values (Ra and Rq) of all three asphalts decrease. This can be attributed to the deepening of the aging process, causing certain components on the asphalt surface to volatilize. As a result, the number of microscopic surface protrusions decreases, leading to a reduction in the roughness value. Currently, different studies on the microstructure of asphalt by various scholars yield inconsistent results [33]. For instance, studies by Koyun, Zhang, and others [28,34] have indicated that the surface roughness of asphalt gradually increases with aging. However, some scholars [33,35] have concluded that surface roughness decreases. Therefore, to further explore the aging characteristics of asphalt during the photothermal oxidative aging process, the molecular weight before and after aging was investigated using GPC.

### 3.3. Aging Characteristics of Low-Grade Asphalt Based on GPC

**Figure 7:**
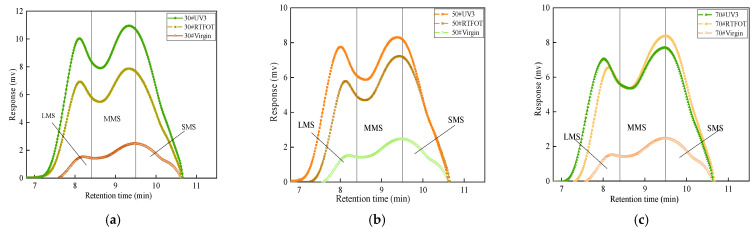
GPC test results of three asphalt: (**a**) 30# asphalt; (**b**) 50# asphalt; (**c**) 70# asphalt.

During the aging process, the polymer present in asphalt can undergo various changes, such as decomposition, shearing, and recombination, leading to alterations in the molecular weight distribution. Gel permeation chromatography (GPC) enables a quantitative evaluation of polymer content and the trend of change in different molecular weight regions. To gain further insights into the performance changes of low-grade asphalt before and after aging, GPC testing was conducted to examine the variation in molecular weight distribution for the three types of asphalt. Molecular weight distribution plays a crucial role in assessing the degree of association among polar components in asphalt binders. In this study, the asphalt molecules were categorized into three regions based on their sizes: large-size molecules (LMS), medium-size molecules (MMS), and small-size molecules (SMS). The test results are illustrated in Figure 7.

**Figure 8:**
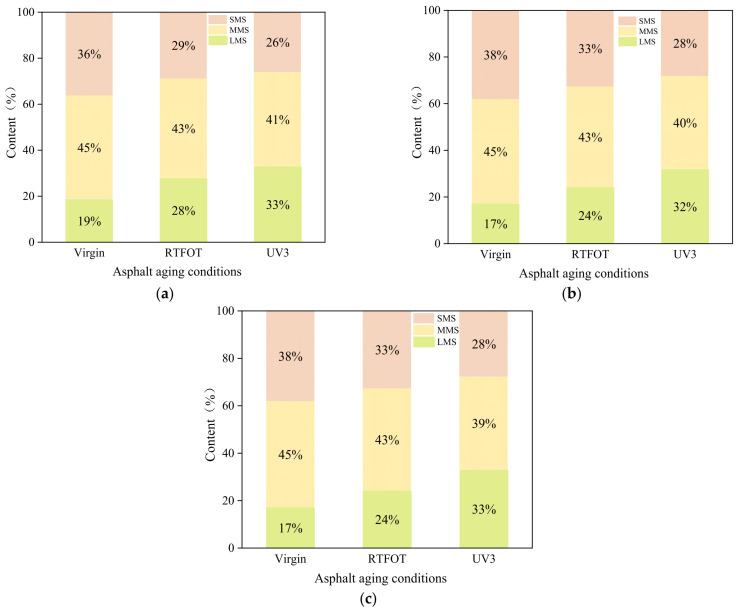
Molecular content of asphalt: (**a**) 30# asphalt; (**b**) 50# asphalt; (**c**) 70# asphalt.

Figure 7 reveals that there is no significant difference in molecular weight among the three asphalts in their unaged state. As the aging process progresses, the three types of asphalt exhibit similar trends, wherein macromolecules gradually form, and some small molecules disappear, transform, or evaporate. This is attributed to the increases in molecular weights of asphaltenes and resins with rising aging temperatures. Given the notable disparity in molecular content among the asphalts, a histogram was employed in this test to quantify and analyze this difference, as depicted in Figure 8. As aging deepens, the LMS percentage of the three asphalts increases, while the SMS and MMS percentages gradually decrease. This indicates that more light components are converted into resins and asphaltenes during the aging process. Figure 8 demonstrates that as the aging progresses from non-aging to ultraviolet aging, the LMS percentages of 30#, 50#, and 70# asphalt are increases of 14, 15, and 16 percentage points, respectively. This suggests that the aging rate of low-grade asphalt is slightly lower than that of 70# asphalt, indicative of better anti-aging ability.

## 4. Rheological Properties of Low-Grade Hard Asphalt

### 4.1. Viscoelastic Properties of Low-Grade Aged Asphalt

**Figure 9:**
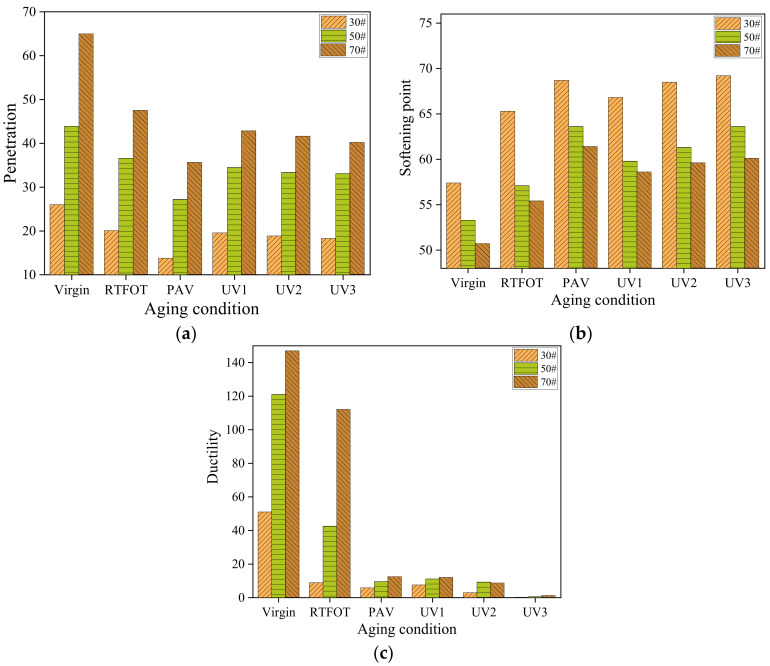
Three indexes of asphalt: (**a**) penetration; (**b**) softening point; (**c**) ductility.

The viscoelastic properties of low-grade aged asphalt were evaluated using penetration, softening point, and ductility tests. The test results are presented in Figure 9. Upon examining Figure 9, it can be observed that the penetration and ductility of all three types of asphalt decrease after aging, while the softening point increases. Furthermore, regardless of thermal oxygen aging or ultraviolet aging, longer aging times correspond to lower penetration and ductility values, as well as higher softening points. This observation aligns with the common understanding that softening points generally increase as penetration decreases [36]. Figure 9a,b indicate that the penetration and softening point of asphalt exhibit minimal changes during the UV aging process from UV1 to UV3. However, there are substantial variations in penetration and softening point between short-term aging and long-term aging in the thermal oxidation stage. Figure 9c demonstrates that both long-term aging and ultraviolet aging considerably reduce the ductility of asphalt. This suggests that prolonged thermal oxygen aging and ultraviolet irradiation significantly diminish the plasticity of asphalt.

### 4.2. DSR Test

**Figure 10:**
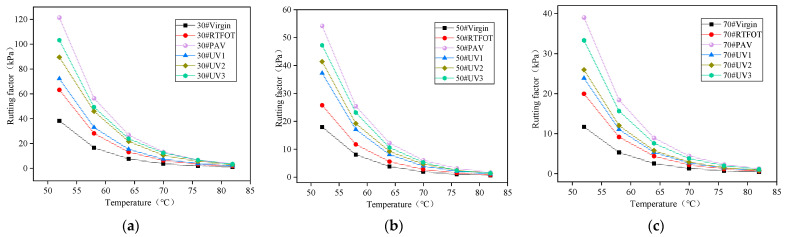
Rutting factor of asphalt: (**a**) 30# asphalt; (**b**) 50# asphalt; (**c**) 70# asphalt.

The rutting factor (G*/sinδ) is a significant indicator of anti-rutting performance, as proposed by the United States SHRP program. A higher rutting factor value indicates better anti-rutting performance, stronger resistance to deformation, and improved high-temperature performance. Figure 10 presents the rutting factors of asphalt samples under various test conditions. The rutting factors of the three types of asphalt exhibit a decreasing trend with increasing temperature, suggesting that higher temperatures lead to reduced shear deformation resistance in asphalt. Particularly within the temperature range of 52 °C to 70 °C, the rutting factor experiences a rapid decrease, indicating that asphalt loses its ability to undergo elastic deformation more rapidly.

Among the different aging conditions, the rutting factor of 30# asphalt is significantly larger than those of the other two types, indicating its strong anti-rutting capability. The order of anti-rutting ability, from highest to lowest, is 30# > 50# > 70#, demonstrating that 70# asphalt has the weakest anti-rutting ability. Furthermore, the anti-rutting ability of asphalt varies under different aging conditions, with overall aging leading to improved rutting factors [37]. Among the different aging methods, PAV aging has the most significant impact on the rutting factor. The ranking of asphalt rutting factor for various aging degrees is as follows: PAV > UV3 > UV2 > UV1 > RTFOT > virgin.

### 4.3. MSCR Test

When evaluating the high-temperature stability of asphalt, the creep recovery rate *R* and the unrecoverable creep compliance *J_nr_* are considered more suitable indicators than the rutting factor. To characterize the high-temperature deformation resistance of asphalt, the rheological properties of 30#, 50#, and 70# asphalt were tested using the MSCR (multi-stress creep recovery) test method. The MSCR test allowed for the assessment of asphalt’s high-temperature performance using two indices: the creep recovery rate *R* and the irreversible creep compliance *J_nr_*. These indices provide valuable insights into the ability of asphalt to recover from deformation and the extent of permanent deformation, respectively.

#### 4.3.1. Creep Recovery Rate

**Figure 11:**
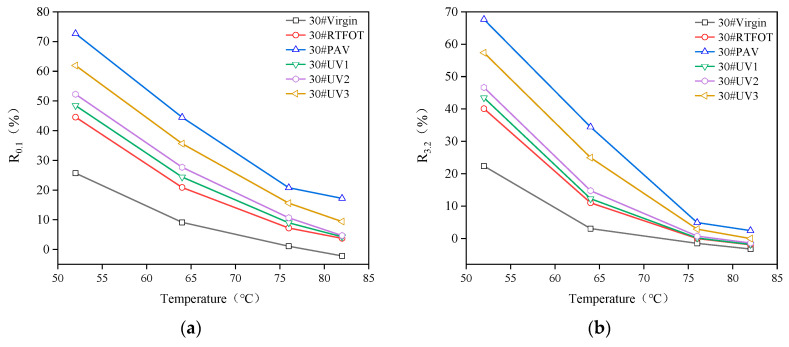

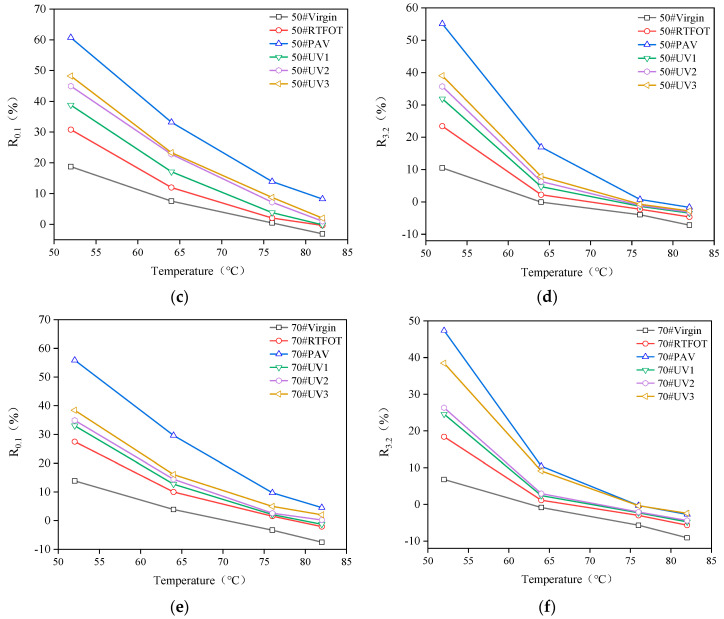
Creep recovery rate of three kinds of asphalt under stress of 0.1 kPa and 3.2 kPa: (**a**) 30#asphalt, *R*_0.1_; (**b**) 30#asphalt, *R*_3.2_; (**c**) 50#asphalt, *R*_0.1_; (**d**) 50#asphalt, *R*_3.2_; (**e**) 70#asphalt, *R*_0.1_; (**f**) 70#asphalt, *R*_3.2_.

Figure 11 displays the creep recovery rate curves of 30#, 50#, and 70# asphalt under stress levels of 0.1 kPa and 3.2 kPa for non-aging, RTFOT aging, PAV aging, and UV aging conditions. Generally, as the temperature increases, the creep recovery rate decreases. By comparing the creep recovery rates under different stress levels, it can be observed that asphalt exhibits a lower creep recovery rate under higher stress, indicating deteriorated creep recovery performance under high-stress conditions. In summary, high stress influences the creep performance of asphalt significantly.

Furthermore, the creep recovery rates of 30# and 50# asphalt are higher than that of 70# asphalt, suggesting that low-grade matrix asphalt demonstrates better creep recovery performance compared to general matrix asphalt. Under the same stress level and temperature conditions, increased aging of asphalt leads to an elevated creep recovery rate. The order of creep recovery rate is PAV > UV3 > UV2 > UV1 > RTFOT > virgin, which aligns with the trend observed for the rutting factor. This indicates that aging promotes the transformation of asphalt viscosity to elasticity, enhancing the elastic ratio and rigidity of the asphalt, thereby improving the creep recovery rate. Aging improves the creep recovery rate of asphalt, enhancing its elasticity and structural stability. Moreover, the creep recovery rates of 30# and 50# asphalt re higher than that of 70# asphalt, indicating that low-grade asphalt exhibits better resistance to rutting compared to regular asphalt.

#### 4.3.2. Unrecoverable Creep Compliance

**Figure 12:**
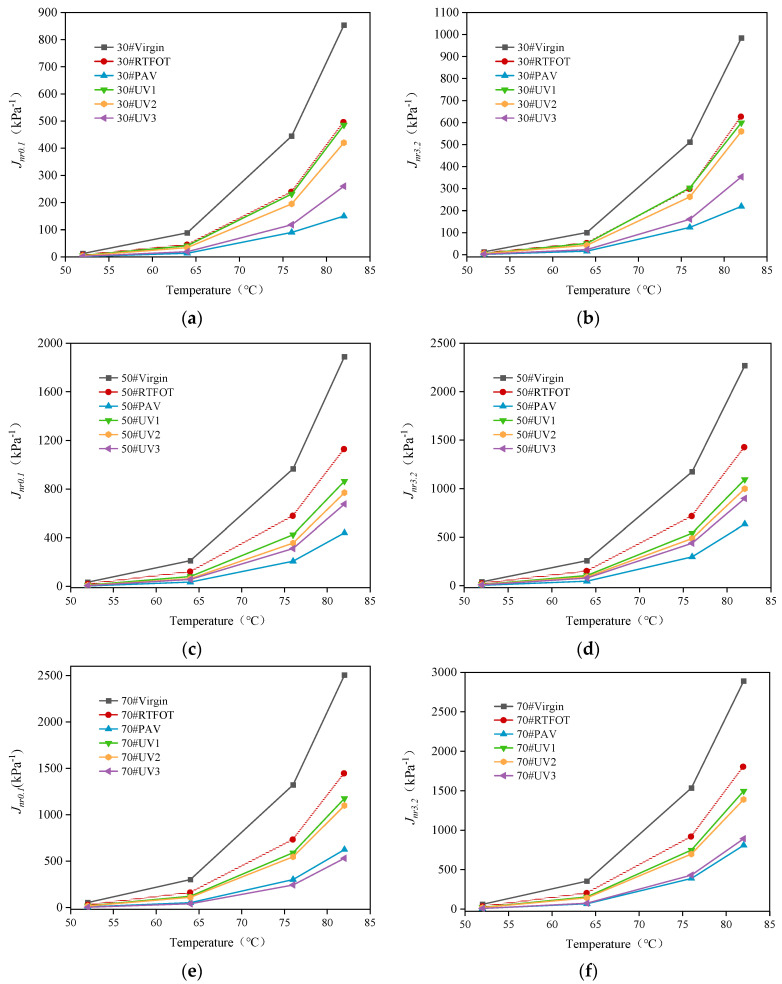
Unrecoverable creep compliance of three kinds of asphalt under stress of 0.1 kPa and 3.2 kPa: (**a**) 30# asphalt, *J_nr_*_0.1_; (**b**) 30# asphalt, *J_nr_*_3.2_; (**c**) 50# asphalt, *J_nr_*_0.1_; (**d**) 50# asphalt, *J_nr_*_3.2_; (**e**) 70# asphalt, *J_nr_*_0.1_; (**f**) 70# asphalt, *J_nr_*_3.2_.

Figure 12 depicts the unrecoverable creep compliance (*J_nr_*) curves of 30#, 50#, and 70# asphalt under stress levels of 0.1 kPa and 3.2 kPa for non-aging, RTFOT aging, PAV aging, and UV aging conditions. Under the same stress level, the *J_nr_* of matrix asphalt increases with the rise in temperature. Moreover, significant changes in *J_nr_* occur in the high-temperature range, while minor variations and flatter curves are observed in the low-temperature range, indicating that high temperature impacts the anti-deformation ability of asphalt substantially.

At the same temperature, *J_nr_* under a stress level of 3.2 kPa is higher than that under a stress level of 0.1 kPa. This suggests that higher stress exerts a greater load on the asphalt, leading to poorer anti-deformation ability. Under the same stress level and temperature, the *J_nr_* of asphalt decreases after aging. This implies that aging reduces the *J_nr_* of asphalt, and the degree of aging has a significant impact on the extent of J_nr_ reduction. The order of unrecoverable creep compliance is PAV < UV3 < UV2 < UV1 < RTFOT < virgin. Photothermal coupling aging falls roughly in the middle position, indicating that the anti-deformation ability of aged asphalt is higher than that of asphalt after RTFOT aging but lower than that of asphalt after PAV aging. With prolonged UV aging time, *J_nr_* gradually decreases, indicating that aging time also affects the resistance to permanent deformation of asphalt [38].

In summary, the *J_nr_* of 70# asphalt is lower than those of 50# and 30# asphalt, indicating inferior high-temperature performance in the light–heat–oxygen coupling environment compared to low-grade asphalt.

## 5. Conclusions

The performance changes and high-temperature rheological properties of 30#, 50#, and 70# asphalt were compared and analyzed through FTIR, GPC, AFM, DSR, and MSCR tests. The effects of thermal oxygen aging and ultraviolet aging on the asphalt were examined, and the aging attenuation law of low-grade asphalt was analyzed and discussed.

(1) The FTIR test results reveal that both thermal oxygen aging and ultraviolet aging lead to the breaking of carbon–carbon double bonds in asphalt, resulting in the formation of polar functional groups, such as carbonyl and sulfoxide groups. The carbonyl index and sulfoxide index of asphalt increase after thermal oxygen aging and ultraviolet aging, with the carbonyl index showing a significant growth rate. In the presence of long-term high ultraviolet radiation, 30# asphalt demonstrates better resistance to aging compared to 50# and 70# asphalt. The AFM elevation image analysis shows an increase in heavy components and surface roughness of asphalt after aging. The GPC test results indicate that during the aging process of asphalt, there is a gradual increase in the LMS% and a decrease in the SMS% and MMS%, indicating the transformation of light components into resin and asphaltene.
(2) The aging process leads to a decrease in the penetration and ductility of asphalt, accompanied by an increase in the softening point. The extent of performance degradation increases with longer aging times. PAV aging has the greatest impact on the basic performance of asphalt, followed by UV aging. Thermal oxygen aging affects the penetration and softening point of asphalt significantly. Long-term thermal oxygen aging and ultraviolet irradiation result in a substantial reduction in the plasticity of asphalt.
(3) The DSR test and MSCR test were conducted to evaluate the rheological properties of asphalt before and after aging. The high-temperature stability of the three types of asphalt is ranked as 30# > 50# > 70#. Notably, 30# matrix asphalt exhibits the highest resistance to deformation. During the photothermal oxidative aging process, low-grade asphalt demonstrates relatively stable stress changes and good high-temperature stability. The rutting factor of 30# asphalt significantly exceeds that of the other types of asphalt during the aging process. Increased temperature, higher stress levels, and prolonged stress duration contribute to a reduced high-temperature stability of asphalt. In terms of rheological properties, the ranking of the same type of asphalt is PAV > UV3 > UV2 > UV1 > RTFOT > virgin. Thermal oxygen aging and ultraviolet aging contribute to the improvement of high-temperature rheological properties of asphalt to some extent.

## Figures and Tables

**Table 1:** The basic parameters of the three asphalts.

Technical Index	30#	50#	70#	Testing Method
Penetration at 25 °C (0.1 mm)	26.0	43.9	60.8	ASTM D5
Softening point (°C)	57.4	53.3	50.7	ASTM D36
Ductility at 15 °C (cm)	22.5	>100	>100	ASTM D113
Dynamic viscosity at 60 °C (Pa·s)	1064.32	353.6	268.8	ASTM D2171

## Data Availability

Data is contained within this article.

## References

[1] Xu Y., Niu K., Zhu H., Chen R., Ou L. (2023). Evaluating the Effects of Polyphosphoric Acid (PPA) on the Anti-Ultraviolet Aging Properties of SBR-Modified Asphalt. Materials.

[2] Liu L., Liu L., Liu Z., Yang C., Pan B., Li W. (2022). Study on the Effect of Ultraviolet Absorber UV-531 on the Performance of SBS-Modified Asphalt. Materials.

[3] Zeng G.D., Wu W.J., Li J.C., Xu Q.S., Li X.H., Yan X.P., Han Y., Wei J.C. (2022). Comparative Study on Road Performance of Low-Grade Hard Asphalt and Mixture in China and France. Coatings.

[4] Wu Y., Zhou X.Y., Wang X.D., Shan L.Y. (2022). Long-Term Service Performance of Hard-Grade Asphalt Concrete Base Pavement Based on Accelerated Loading Test of Full-Scale Structure. Sustainability.

[5] Tong Y.J., Shen B.X., Liu J.C., Zhao J.G. (2017). Characterization of hard-grade asphalt using entropy analysis. Pet. Sci. Technol..

[6] Tong Y.J., Shen B.X., Liu J.C., Yao Z.T., Fang W.F. (2017). Preparation and evaluation of 30# hard grade asphalt. Pet. Sci. Technol..

[7] Jiang W., Yuan D.D., Shan J.H., Ye W.L., Lu H.H., Sha A.M. (2022). Experimental study of the performance of porous ultra-thin asphalt overlay. Int. J. Pavement Eng..

[8] Sha A., Liu Z., Jiang W., Qi L., Hu L., Jiao W., Barbieri D.M. (2021). Advances and development trends in eco-friendly pavements. J. Road Eng..

[9] Zhang F., Cao Y., Sha A., Lou B., Song R., Hu X. (2022). Characterization of asphalt mixture using X-ray computed tomography scan technique after freeze-thaw cycle and microwave heating. Constr. Build. Mater..

[10] Yang E., Ma B., Li A., Ren D., Qiu Y. (2022). Effect of short-term aging on high temperature performance of expansion joint binder. Munic. Eng. Technol..

[11] Li R., Wang P.Z., Xue B., Pei J.Z. (2015). Experimental study on aging properties and modification mechanism of Trinidad lake asphalt modified bitumen. Constr. Build. Mater..

[12] Cao Z.L., Huang X.Q., Yu J.Y., Han X.B., Wang R.Y., Li Y. (2020). Study on all-components regeneration of ultraviolet aged SBS modified asphalt for high-performance recycling. J. Clean. Prod..

[13] Ye Q., Dong W., Wang S., Li H. (2020). Research on the Rheological Characteristics and Aging Resistance of Asphalt Modified with Tourmaline. Materials.

[14] Kolodziej K., Bichajlo L., Siwowski T. (2021). Effects of Aging on the Physical and Rheological Properties of Trinidad Lake Asphalt Modified Bitumen. Materials.

[15] Hamid A., Baaj H., El-Hakim M. (2022). Rutting Behaviour of Geopolymer and Styrene Butadiene Styrene-Modified Asphalt Binder. Polymers.

[16] Anochie-Boateng J.K., Malisa J.T. (2023). A Case Investigation into Causes of Premature Rutting Failures in Rehabilitated Asphalt Pavements in Tanzania. J. Test. Eval..

[17] Wang H., Zhu Y., Zhang W., Shen S., Wu S., Mohammad L.N.N., She X. (2023). Effects of Field Aging on Material Properties and Rutting Performance of Asphalt Pavement. Materials.

[18] Nciri N., Kim N., Cho N. (2021). Spent Graphite from End-of-Life Lithium-Ion Batteries (LIBs) as a Promising Nanoadditive to Boost Road Pavement Performance. Materials.

[19] Yuan D., Jiang W., Sha A., Xiao J., Wu W., Wang T. (2023). Technology method and functional characteristics of road thermoelectric generator system based on Seebeck effect. Appl. Energy.

[20] Zhang F., Cao Y., Sha A., Wang W., Song R., Lou B. (2022). Mechanism, rheology and self-healing properties of carbon nanotube modified asphalt. Constr. Build. Mater..

[21] Sultana S., Bhasin A. (2014). Effect of chemical composition on rheology and mechanical properties of asphalt binder. Constr. Build. Mater..

[22] Mirwald J., Werkovits S., Camargo I., Maschauer D., Hofko B., Grothe H. (2020). Investigating bitumen long-term-ageing in the laboratory by spectroscopic analysis of the SARA fractions. Constr. Build. Mater..

[23] Liu N., Liu L., Li M., Sun L. (2023). Effects of zeolite on rheological properties of asphalt materials and asphalt-filler interaction ability. Constr. Build. Mater..

[24] Nivitha M.R., Prasad E., Krishnan J.M. (2016). Ageing in modified bitumen using FTIR spectroscopy. Int. J. Pavement Eng..

[25] Nagabhushanarao S.S., Vijayakumar A.S. (2021). Chemical and rheological characteristics of accelerate aged asphalt binders using rolling thin film oven. Constr. Build. Mater..

[26] Feng Z.G., Bian H.J., Li X.J., Yu J.Y. (2016). FTIR analysis of UV aging on bitumen and its fractions. Mater. Struct..

[27] Yang Z., Zhang X.N., Zhang Z.Y., Zou B.J., Zhu Z.H., Lu G.Y., Xu W., Yu J.M., Yu H.Y. (2018). Effect of Aging on Chemical and Rheological Properties of Bitumen. Polymers.

[28] Zhang H.L., Yu J.Y., Feng Z.G., Xue L.H., Wu S.P. (2012). Effect of aging on the morphology of bitumen by atomic force microscopy. J. Microsc..

[29] Zeng W.B., Wu S.P., Pang L., Chen H.H., Hu J.X., Sun Y.H., Chen Z.W. (2018). Research on Ultra Violet (UV) aging depth of asphalts. Constr. Build. Mater..

[30] Ma F., Wang Y.J., Fu Z., Tang Y.J., Dai J.S., Li C., Dong W.H. (2022). ARTICLE INFO Keywords: Natural rock asphalt FTIR spectroscopy Ageing mechanism Carbonyl index Sulfoxide index. Constr. Build. Mater..

[31] Pasetto M., Baliello A., Giacomello G., Pasquini E. (2023). Advances in the Rheology of Synthetic Binder for Sustainable Road Pavements: An Improved Protocol for DSR Testing. Sustainability.

[32] Qian Y., Guo F., Leng Z., Zhang Y., Yu H.Y. (2020). Simulation of the field aging of asphalt binders in different reclaimed asphalt pavement (RAP) materials in Hong Kong through laboratory tests. Constr. Build. Mater..

[33] Wang M., Liu L.P. (2017). Investigation of microscale aging behavior of asphalt binders using atomic force microscopy. Constr. Build. Mater..

[34] Koyun A., Buchner J., Wistuba M.P., Grothe H. (2022). Rheological, spectroscopic and microscopic assessment of asphalt binder ageing. Road Mater. Pavement Des..

[35] Xing C.W., Liu L.P., Li M.C. (2020). Chemical Composition and Aging Characteristics of Linear SBS Modified Asphalt Binders. Energy Fuels.

[36] Zhang Z.P., Chen L.Q., Peng J., Sun J., Zhang D.L., Li X., Wen F.S., Liu H. (2022). Preparation and properties of a novel high-viscosity modified bitumen. Constr. Build. Mater..

[37] Wu S.H., He R., Chen H.X., Li W.K., Li G.H. (2020). Rheological Properties of SBS/CRP Composite Modified Asphalt under Different Aging Treatments. Materials.

[38] Kleiziene R., Panasenkiene M., Vaitkus A. (2019). Effect of Aging on Chemical Composition and Rheological Properties of Neat and Modified Bitumen. Materials.

